# Simultaneous ileocolic and abdominoperineal resection using the da Vinci SP system for synchronous triple colorectal cancer: a case report

**DOI:** 10.1093/jscr/rjaf565

**Published:** 2025-07-28

**Authors:** Hiroto Tanaka, Yasutake Uchima, Daijiro Kagawa, Keishiro Esaki, Kenta Hikotani, Tomoki Henna, Yuta Murakami, Yuto Tedokon, Hiroaki Kawamoto, Yoshiyuki Nakasone, Yasukazu Ikehara, Tuyoshi Tamae

**Affiliations:** Department of Gastroenterologic Surgery, Chubo Tokushukai Hospital, 801 Higa, Kitanakagusuku-son, Nakagami-gun, Okinawa-ken 901-2393, Japan; Department of Gastroenterologic Surgery, Chubo Tokushukai Hospital, 801 Higa, Kitanakagusuku-son, Nakagami-gun, Okinawa-ken 901-2393, Japan; Department of Gastroenterologic Surgery, Chubo Tokushukai Hospital, 801 Higa, Kitanakagusuku-son, Nakagami-gun, Okinawa-ken 901-2393, Japan; Department of Gastroenterologic Surgery, Chubo Tokushukai Hospital, 801 Higa, Kitanakagusuku-son, Nakagami-gun, Okinawa-ken 901-2393, Japan; Department of Gastroenterologic Surgery, Chubo Tokushukai Hospital, 801 Higa, Kitanakagusuku-son, Nakagami-gun, Okinawa-ken 901-2393, Japan; Department of Gastroenterologic Surgery, Chubo Tokushukai Hospital, 801 Higa, Kitanakagusuku-son, Nakagami-gun, Okinawa-ken 901-2393, Japan; Department of Gastroenterologic Surgery, Chubo Tokushukai Hospital, 801 Higa, Kitanakagusuku-son, Nakagami-gun, Okinawa-ken 901-2393, Japan; Department of Gastroenterologic Surgery, Chubo Tokushukai Hospital, 801 Higa, Kitanakagusuku-son, Nakagami-gun, Okinawa-ken 901-2393, Japan; Department of Gastroenterologic Surgery, Chubo Tokushukai Hospital, 801 Higa, Kitanakagusuku-son, Nakagami-gun, Okinawa-ken 901-2393, Japan; Department of Gastroenterologic Surgery, Chubo Tokushukai Hospital, 801 Higa, Kitanakagusuku-son, Nakagami-gun, Okinawa-ken 901-2393, Japan; Department of Gastroenterologic Surgery, Chubo Tokushukai Hospital, 801 Higa, Kitanakagusuku-son, Nakagami-gun, Okinawa-ken 901-2393, Japan; Department of Gastroenterologic Surgery, Chubo Tokushukai Hospital, 801 Higa, Kitanakagusuku-son, Nakagami-gun, Okinawa-ken 901-2393, Japan

**Keywords:** robot-assisted surgery, da Vinci SP system, single-port surgery, colorectal cancer, simultaneous resection, minimally invasive surgery

## Abstract

Robot-assisted surgery for colorectal cancer is gaining traction in Japan, particularly with the approval of the da Vinci SP system for insurance coverage in 2022. This system enables precise and flexible procedures through a single port accommodating three instrument arms and a camera. We present a case involving a 73-year-old man with synchronous tumors in the cecum, sigmoid colon, and rectum (Rb). Simultaneous resection was successfully performed using the da Vinci SP system by a robotic surgical team and a perineal team without complications. To our knowledge, this is the first reported case of simultaneous resection of triple colorectal lesions using this system. The single-port design and 360° rotating arms enhanced maneuverability and reduced the need for multiple incisions compared to conventional platforms. The da Vinci SP system shows promise for treating multi-lesion colorectal cancer cases, though further studies are required to validate its long-term efficacy and broader clinical utility.

## Introduction

Laparoscopic surgery has been the mainstream minimally invasive approach for gastrointestinal procedures since its first report by Jacobs *et al*. [1]. However, robot-assisted surgery has emerged at the forefront of surgical innovation. In Japan, the adoption of robot-assisted surgery for colorectal cancer has grown rapidly, particularly following its approval for insurance coverage [2]. Robotic surgery offers several advantages over conventional laparoscopic methods, including tremor filtration, multi-jointed instruments, and motion scaling, thereby enabling more precise and delicate surgical maneuvers. Despite these technical advantages, current clinical evidence remains limited regarding the superiority of robotic surgery over laparoscopic surgery in rectal and colon cancer cases, particularly in terms of long-term outcomes [3–5]. Further technological refinement and clinical validation are necessary.

The da Vinci SP system (Intuitive Surgical Inc., CA, USA), introduced in the United States in 2018 and approved for insurance coverage in Japan in September 2022, represents a fourth-generation robotic platform. Its key feature is the ability to manipulate three instrument arms and a flexible endoscopic camera through a single 2.5 cm port, enabling highly precise and ergonomic surgery in confined spaces.

Herein, we present the world’s first case of synchronous triple colorectal cancer—comprising tumors in the cecum, sigmoid colon, and rectum—resected simultaneously using the da Vinci SP system.

## Case report

A 73-year-old man with a body mass index of 22.6 kg/m^2^ and no significant past medical history presented with bowel movement irregularities and unintentional weight loss. Colonoscopy revealed three distinct lesions: a laterally spreading tumor in the cecum, a semi-circumferential type 2 lesion in the sigmoid colon, and another semi-circumferential type 2 lesion in the lower rectum (Rb). Biopsies confirmed a cecal adenoma, a mucinous adenocarcinoma in the sigmoid colon, and a moderately differentiated adenocarcinoma in the rectum.

**Figure 1:**
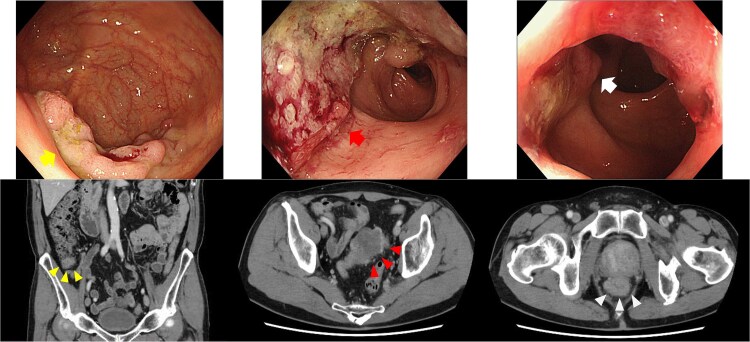
Endoscopy and enhanced CT endoscopy showed tumors in the cecum (LST-G, upper left), sigmoid (type 2, upper middle), and rectum (type 2, upper right). Enhanced CT provided important insights into the wall thickness in the cecum (lower left), sigmoid colon (lower middle), and rectum (lower right).

Contrast-enhanced computed tomography (CT) demonstrated bowel wall thickening in all three sites, with regional lymphadenopathy observed in the sigmoid and rectal mesenteries. No distant metastasis was identified (Fig. 1). Given the proximity of the rectal tumor to the anal verge (within 1 cm), sphincter preservation was deemed infeasible, and abdominoperineal resection was planned. Although endoscopic resection was initially considered for the cecal lesion, surgical resection was chosen following multidisciplinary consultation.

**Figure 2:**
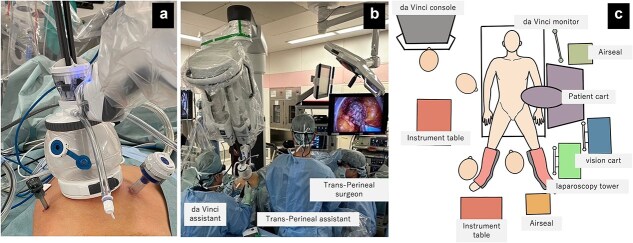
Field of view and positioning of the assistant. (a) da Vinci SP docked to access port with two assistant ports: 12 mm trocar on the left side and 5 mm trocar on the right side. (b) da Vinci SP with trans-perineal endoscopic approach, (c) Operation room set up and positioning.

Under general anesthesia, the patient was placed in the lithotomy position. A certified da Vinci Xi proctor served as the console surgeon for the da Vinci SP system, while a perineal team conducted a simultaneous transperineal laparoscopic approach (Fig. 2). A 4-cm umbilical incision was made for placement of the SP access port. Additional assistant ports (a 12-mm trocar on the left and a 5-mm trocar on the right) were inserted. Due to limited visibility caused by robotic arms obstructing the assistant’s view, a 3D head-mounted display system (Weaps; Hogy Medical Co., LTD, Tokyo, Japan) was employed.

**Figure 3:**
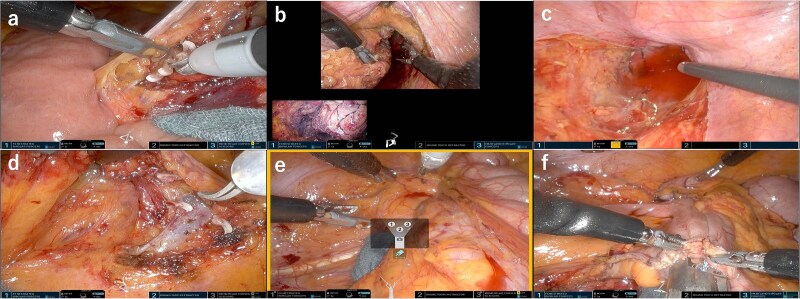
Field of view of the console. (a) Medial approach for sigmoid colon and clipped and ligated IMA. (b) Rectum was mobilized passively upon rendezvous with the transperineal approach. (c) Total mesorectal excision was performed in cooperation with the perineal approach while preserving the pelvic splanchnic nerves, (d) Medial approach for ileocecal resection and ligated ileocecal artery and vein. (e) Lateral approach for ileocecal resection using relocate mode. (f) Intracorporeal anastomosis.

The sigmoid and rectal resections followed a medial-to-lateral approach. Dissection was performed around the root of the inferior mesenteric artery (IMA), followed by D3 lymphadenectomy using bipolar coagulation. The IMA and inferior mesenteric vein were clipped and divided at the same level (Fig. 3a and b). The descending colon was mobilized laterally (Fig. 3c), and total mesorectal excision was performed. The dissection reached the pelvic floor, where rendezvous with the perineal team was achieved at the S4 level. Neurovascular bundles were preserved through careful dissection along Denonvilliers’ fascia and the anterior rectal wall (Fig. 3d–f).

The patient was then repositioned in a left-tilted Trendelenburg position for the ileocolic resection. The ileocolic vessels were identified and clipped, with D3 lymphadenectomy performed (Fig. 3g). The right colon was mobilized and transected using a linear stapler (Fig. 3h). Reconstruction was performed using an intracorporeal side-to-side overlap anastomosis (Fig. 3i). Specimen retrieval was conducted through the umbilical incision. A pelvic drain was placed via the right port, and a stoma was fashioned through the left port.

The total operative time was 370 min, including 297 min of console time. The estimated blood loss was 50 mL. The patient resumed oral intake on postoperative day 4, received stoma care education, and was discharged without complications on postoperative day 14.

**Figure 4:**
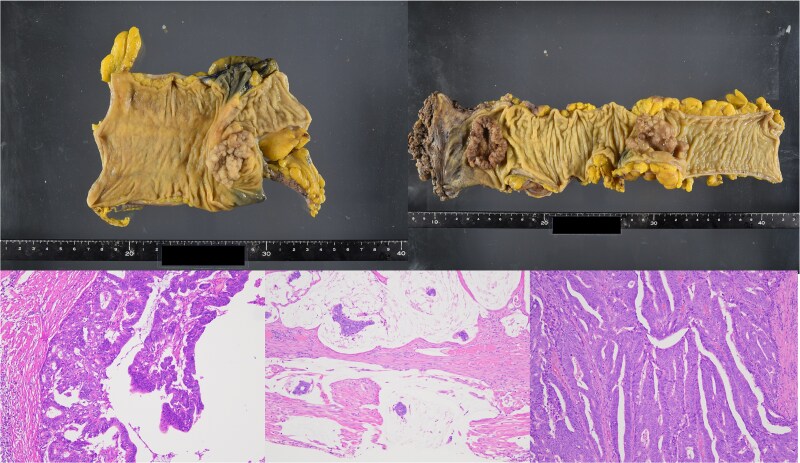
Resected specimen and Hematoxylin and eosin (H&E) staining. (a) Resected specimen from the cecum. (b) Resected specimens from sigmoid colon and rectum. (c) H&E staining of cecum (×100) b: Sigmoid (×100) c: Rectum (×100).

Histopathology revealed a moderately differentiated adenocarcinoma in the cecum (pT3, pN1a, cM0), a mucinous adenocarcinoma in the sigmoid colon (pT4a, pN2a, cM0), and a moderately differentiated adenocarcinoma in the rectum (pT3, pN0, cM0) (Fig. 4).

## Discussion

To our knowledge, this is the first reported case of synchronous resection of triple colorectal cancer using the da Vinci SP system. Most robotic systems currently available are multiport platforms, and although their port placement has become more streamlined over time, incorrect positioning may still result in interference between robotic arms or docking difficulties [6]. For complex or extensive procedures, including multiple organ resections, the conventional multiport approach may necessitate additional incisions, port reconfiguration, and may result in decreased surgical efficiency.

In contrast, the da Vinci SP system, with its single-port design, eliminates the need for multiple incisions. Its ‘relocate’ mode allows 360° instrument rotation without requiring redocking. Furthermore, the cobra-mode camera provides versatile cranial-caudal viewing angles [7]. These features were particularly advantageous in the present case, which involved two separate extensive dissections. By fully utilizing the articulating arms and access port kit, the working space was maximized to 6.5 cm without wound interference, enhancing operative efficiency and reducing the need for assistant intervention.

Emerging reports suggest that SP-assisted pelvic surgeries may offer superior short-term outcomes over laparoscopy [8]. Comparative studies have also noted reduced incision lengths, lower pain scores, and shorter hospital stays with SP versus da Vinci Xi systems [9]. Nevertheless, these findings are based on retrospective data; high-quality prospective trials and long-term follow-up are still required.

Single-incision laparoscopic surgery (SILS) was first introduced for colorectal cancer in 2008 [10]. While studies later demonstrated its non-inferiority in terms of oncologic safety and perioperative outcomes [11–13], its technical complexity limited widespread adoption. In this regard, the da Vinci SP system offers an attractive alternative by addressing key limitations of SILS—namely instrument crowding and tremor control—thus shortening the learning curve and improving reproducibility.

In the present case, operative time was prolonged, possibly due to the institutional learning curve associated with SP adoption. Additionally, the da Vinci SP system currently lacks proprietary stapling or energy devices, necessitating assistant cooperation for these tasks. Ongoing technological development may alleviate this dependency in future iterations.

In conclusion, simultaneous resection of triple colorectal cancer lesions using the da Vinci SP system was technically feasible and safe. This case demonstrates the system’s potential in complex colorectal procedures, offering enhanced maneuverability and minimal invasiveness. Further case accumulation and clinical studies are warranted to assess long-term outcomes and broaden the scope of SP application in colorectal surgery.
